# Fluxes of Water through Aquaporin 9 Weaken Membrane-Cytoskeleton Anchorage and Promote Formation of Membrane Protrusions

**DOI:** 10.1371/journal.pone.0059901

**Published:** 2013-04-03

**Authors:** Thommie Karlsson, Anastasia Bolshakova, Marco A. O. Magalhães, Vesa M. Loitto, Karl-Eric Magnusson

**Affiliations:** 1 Division of Medical Microbiology, Department of Clinical and Experimental Medicine, Faculty of Health Sciences, Linkoping University, Linkoping, Sweden; 2 Faculty of Dentistry, University of Toronto, Toronto, Canada; Emory University School of Medicine, United States of America

## Abstract

All modes of cell migration require rapid rearrangements of cell shape, allowing the cell to navigate within narrow spaces in an extracellular matrix. Thus, a highly flexible membrane and a dynamic cytoskeleton are crucial for rapid cell migration. Cytoskeleton dynamics and tension also play instrumental roles in the formation of different specialized cell membrane protrusions, viz. lamellipodia, filopodia, and membrane blebs. The flux of water through membrane-anchored water channels, known as aquaporins (AQPs) has recently been implicated in the regulation of cell motility, and here we provide novel evidence for the role of AQP9 in the development of various forms of membrane protrusion. Using multiple imaging techniques and cellular models we show that: (i) AQP9 induced and accumulated in filopodia, (ii) AQP9-associated filopodial extensions preceded actin polymerization, which was in turn crucial for their stability and dynamics, and (iii) minute, local reductions in osmolarity immediately initiated small dynamic bleb-like protrusions, the size of which correlated with the reduction in osmotic pressure. Based on this, we present a model for AQP9-induced membrane protrusion, where the interplay of water fluxes through AQP9 and actin dynamics regulate the cellular protrusive and motile activity of cells.

## Introduction

Cell migration requires tightly regulated membrane dynamics and cytoskeleton remodeling to allow for rapid shape change and navigation through the extracellular matrix (ECM) of different tissues. It also depends on a variety of other factors, such as the availability of adhesion receptors and substratum composition, tension and dimensionality [1]–[3]. Although, several distinct modes of cell migration have been described [4]–[11], they all employ formation of specialized membrane protrusions, i.e. filopodia, lamellipodia and blebs.

Filopodia, which are tightly associated with activation of the small GTPase Cdc42 [12], usually protrude from the lamellipodium. They are characteristically long finger-like projections within which the actin filaments are tightly bundled, and are thought to function as gradient sensors to orient the migrating cell [13]–[16] and to provide traction force [17], [18] through adhesion proteins [19]. Moreover, a specific set of proteins give them a unique character; Ena/VASP proteins preventing capping of the polymerizing barbed ends [20]–[22], myosin X transporting cargo like Mena/VASP [23] to the filopodial tips [24], IRSp53 deforming the membrane through its inverse BAR (I-BAR) domain [25], fascin cross-linking actin filaments [26], [27] and formins like mDia2 promoting polymerization of long unbranched actin filaments [28]–[30]. Still, the molecular mechanisms and signaling pathways involved in filopodial induction are not fully understood [30]. In the convergent elongation model, Svitkina and co-workers [31] proposed that it occurs through reorganization of the Arp2/3-mediated dendritic network in lamellipodia [31], where privileged actin filaments within the branched lamellipodial network associate with formins, Ena/VASP and fascin. In the tip-nucleation model, plasma membrane-associated formins nucleate actin filaments, which can explain the appearance of filopodia upon knock-down of the Arp2/3 complex and other lamellipodium-associated proteins [32]. Still, it is debated which of the models being most relevant [31]–[33]. It is generally assumed that extensions of filamentous actin pushes the membrane through a Brownian-ratchet mechanism [34], [35], but membrane-deforming proteins and fluxes of water have also been proposed to help generate such protrusions [36]–[39]. Moreover, bleb formation has recently been implicated in cell motility [8], [11], being induced by an increased hydrostatic pressure and not requiring actin polymerization to expand [40]–[42]. The tension of the cortical actin cytoskeleton has been assumed to increase the local pressure and initiate a bleb, which in turn may neutralize the pressure by allowing fluid to flow freely into the bleb from the poro-elastic, gel-like cytoplasm [42], [43]. Such bleb-based motile behavior has been observed for cells migrating in 3D matrices [6], [11], [44].

Aquaporins (AQPs) are membrane-anchored water channels [45], [46], defined by their permeability characteristics; the aquaporins, are solely permeable to water and the aquaglyceroporins allow both water and some neutral solutes like glycerol to pass [47]. Pivotal roles have been attributed to AQPs in the regulation of cell motility and morphology, where AQP9 has been shown to localize to the leading edge in migrating neutrophils [37], [39], [48], [49]. Moreover, Loitto and co-workers [38], showed that overexpression of AQP9 induced a filopodial phenotype in fibroblasts, a trait that was later confirmed for neutrophils [50]. Hypothetically, polarized expression of AQP9 and increased hydrostatic pressure at the site of water influx could push the membrane forward and thereby create space and availability of G-actin for actin polymerization [39].

The aim of the present study was to elucidate the mechanisms behind membrane protrusions, and specifically the interplay between AQP9 and actin cytoskeleton dynamics. Since mammalian cells often express two or more AQPs and knock-down of one channel may result in upregulation of another, we used HEK-293 cells as a model system, in which we overexpressed and visualized GFP-AQP9 together with other cytoskeletal probes. Here, we provide new evidence that AQP9 not only induced highly dynamic filopodia, but also accumulated in the membrane before bleb formation. Moreover, AQP9-rich filopodial elongations were initially devoid of filamentous actin. We therefore propose that localized accumulation of AQP9 and influx of water help increase the hydrostatic pressure and space between the membrane and the cortical actin cytoskeleton, whereby barbed ends of actin filaments are exposed to G-actin and further elongation is enabled. The influx of water into the dense, gel-like cytoplasm should also facilitate diffusion of G-actin monomers to the fast-growing barbed ends. In addition, local osmotic changes yielded more filopodial bleb-like protrusion in cells expressing AQP9, suggesting that filopodia can play a potential role as sensors protecting the cell from general osmotic stress.

In summary, we show that filopodia, and bleb induction are facilitated by localized accumulation of AQP9 and influx of water, providing a basis for filopodia and lamellipodia formation.

## Results

### AQP9 Induces Filopodia in HEK-293 Cells

**Figure 1:**
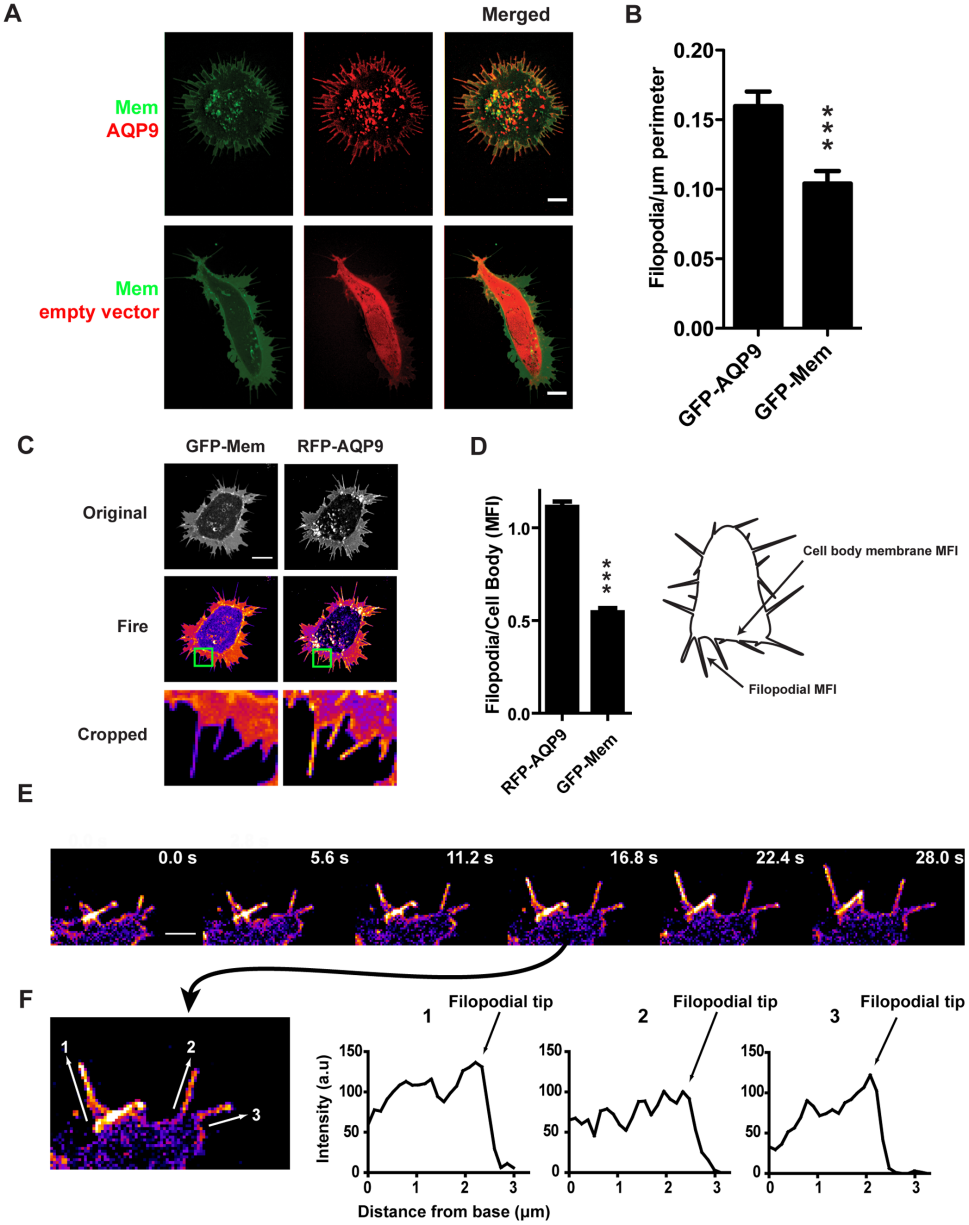
Characterization of AQP9-induced filopodia. (A) Representative confocal images of HEK-293 cells transfected with tagRFP-AQP9 or empty vector together with GFP-Mem to label the membrane. Intensities have been adjusted linearly to visualize the relative expression and localization of both fluorophores. Scalebar 10 µm. (B) Quantification of peripheral filopodia in HEK-293 cells transfected with GFP-AQP9 or GFP-Mem. The data is presented as mean number of filopodia/µm perimeter(±SEM; n = 34–43 cells/group). (C) Representative confocal images of HEK-293 cells transfected with tagRFP-AQP9 and GFP-Mem. Images are pseudo-colored in fire scale to visualize the differences between the two vectors in the filopodia. The intensities have been adjusted linearly to visualize the relative distribution of both fluorophores. The lower panel represents enlargement of the green box. Scalebar 10 µm. (D) Ratiometric measurements of mean fluorescence intensity (MFI) in the filopodial membrane divided by MFI in the cell body membrane in HEK-293 cells transfected with both tagRFP-AQP9 and GFP-Mem. Measurement areas are illustrated in the schematic image. The data is presented as mean (± SEM, n = 51 filopodia/group). (E) Montage of a representative confocal time-lapse of a HEK-293 cell overexpressing GFP-AQP9 pseudo-colored in fire scale to visualize AQP9 localization in growing filopodia. The linear intensity has been adjusted to visualize differences in fluorescent intensity. Scalebar 2 µm. (F, left panel) An enlarged image from (E) showing the points of measurements for the profile plots presented in the right panel. (F, right panel) Intensity profile plots of filopodia during growth to visualize AQP9 accumulation in filopodial tips.

To confirm a previous observation that the presence of AQP9 can yield a filopodial phenotype [38], we expressed fluorescently tagged AQP9 in HEK-293 cells and visualized the protein with live-cell confocal microscopy. Indeed, tagRFP-AQP9 localized to the plasma membrane and induced a filopodial phenotype as evident from control transfections with an empty tagRFP vector and a GFP-membrane vector (GFP-Mem; Fig. 1A). This was further confirmed by quantification of peripheral filopodia/µm perimeter (P<0.0005, Students T-test, n = 34–43 cells, Fig. 1B) where GFP-AQP9- and GFP-Mem-transfected cells displayed a mean of 0.16 (±0.01) and 0.10 (±0.01) filopodia/µm (mean± SEM) respectively. Incidentally, the number of filopodia/µm perimeter decreased from 0.16 (±0.01) to 0.1 (±0.01) and 0.05 (±0.04; mean± SEM; p<0.05, n = 6–43 cells/group) after inhibiting AQP9 with 10 or 100 µM HgCl_2_, respectively.

Accumulation of AQP9 in filopodia was not an effect of membrane folding (Fig. 1C), as evidenced by the ratios of the mean fluorescence intensity (MFI) of filopodia and MFI of cell body membrane (Fig. 1D, n = 51 filopodia; the ratio was for AQP9 1.11±0.03, and for Mem 0.54±0.02; mean ± SEM). Moreover, the localization of AQP9 in filopodia was confirmed by immuno-gold labeling and electron microscopy (data not shown). As revealed by imaging, the filopodia were highly dynamic and constantly protruding, retracting and moving laterally along the cell body (Fig. 1E, Video S1, upper panel). The dynamic feature decreased instantly upon treatment with 1 µM Hg^2+^ and disappeared completely with 10 µM Hg^2+^ (Video S2), which is 30 -fold less than what is generally used to block AQP channels [51]. In addition, we observed a tendency of AQP9 to accumulate at the tip of the filopodia during elongation, as indicated by the time series (Fig. 1E) and the intensity profile plots (Fig. 1F). This was only visible during expansion of filopodia and disappeared during termination of elongation.

### Myosin X and IRSp53 Localize in AQP9-induced Filopodia

**Figure 2:**
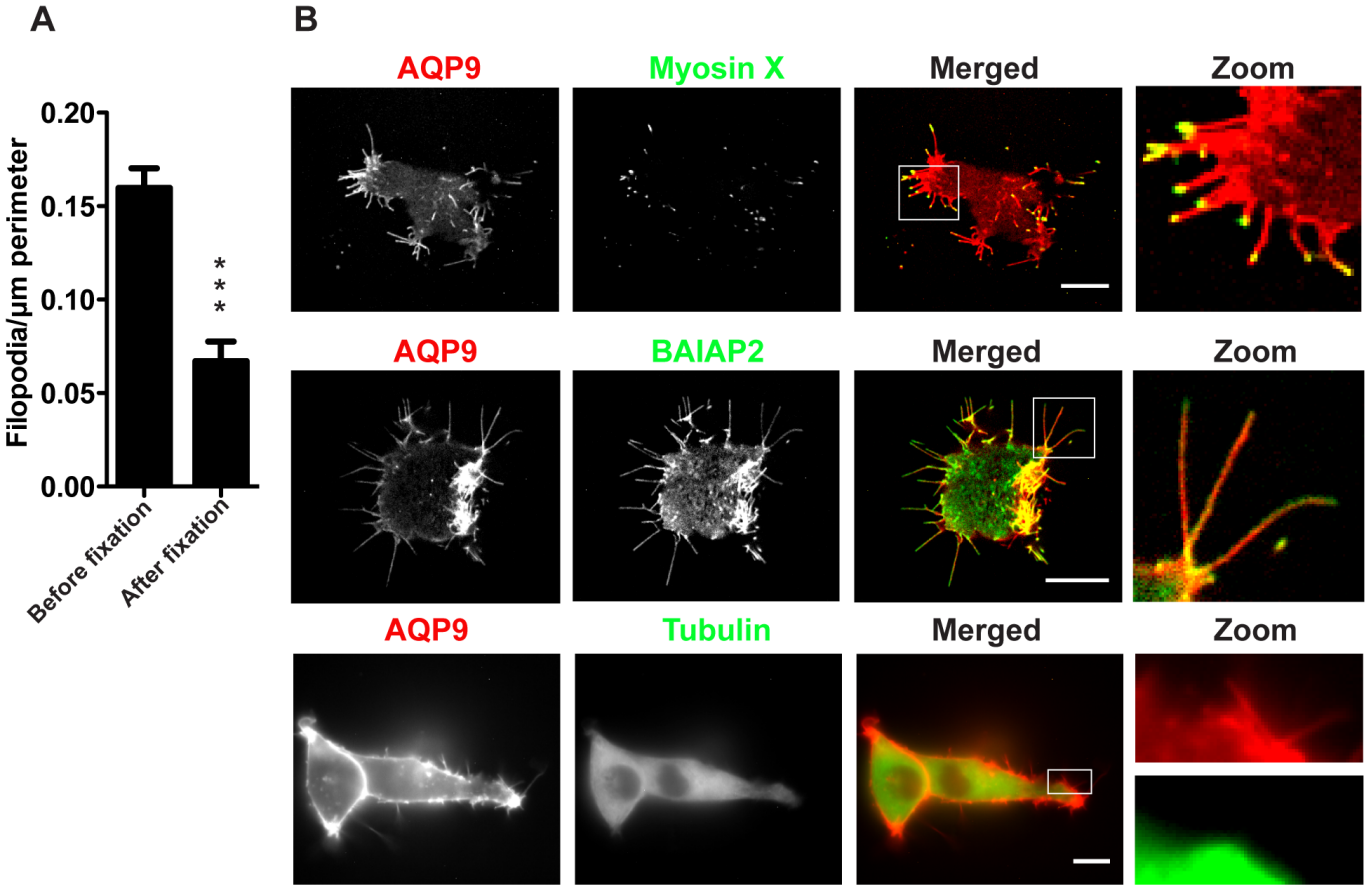
Localization of tubulin, myosin X and BAIAP2 in GFP-AQP9-transfected HEK-293 cells. (A) Quantification of peripheral filopodia in GFP-transfected HEK-293 cells before and after fixation. Data are presented as mean (± SEM, n = 12–43 cells/group). (B) Images captured at the basal part of a HEK-293 cells co-expressing AQP9 and other filopodia-associated proteins fused to GFP or tagRFP. Linear intensities have been adjusted to visualize the relative distribution of both fluorophores. The zoom panel illustrating AQP9 and tubulin is split to emphasize the lack of tubulin in filopodia. Scalebar 10 µm.

Since filopodia are thin and fragile structures we assessed the effects of fixation, which showed that more than half of them were lost during fixation (Fig. 2A, n = 12–43 cells/group, p<0.0001), i.e. before fixation they were 0.16 (±0.01) and after fixation 0.07 (±0.01; mean±SEM) filopodia/µm perimeter, respectively. Thus, due to their fragile nature, fluorescently tagged protein probes had to be used instead of immuno-labeling. When expressing established filopodial markers together with AQP9, both myosin X and brain-specific angiogenesis inhibitor protein 2 (BAIAP2; a human homologue to IRSp53 [52] localized to these filopodia, whereas tubulin did not (Fig. 2B).

### AQP9-induced Filopodia Respond to Hypo-osmotic Changes by Developing Bleb-like Protrusions

**Figure 3:**
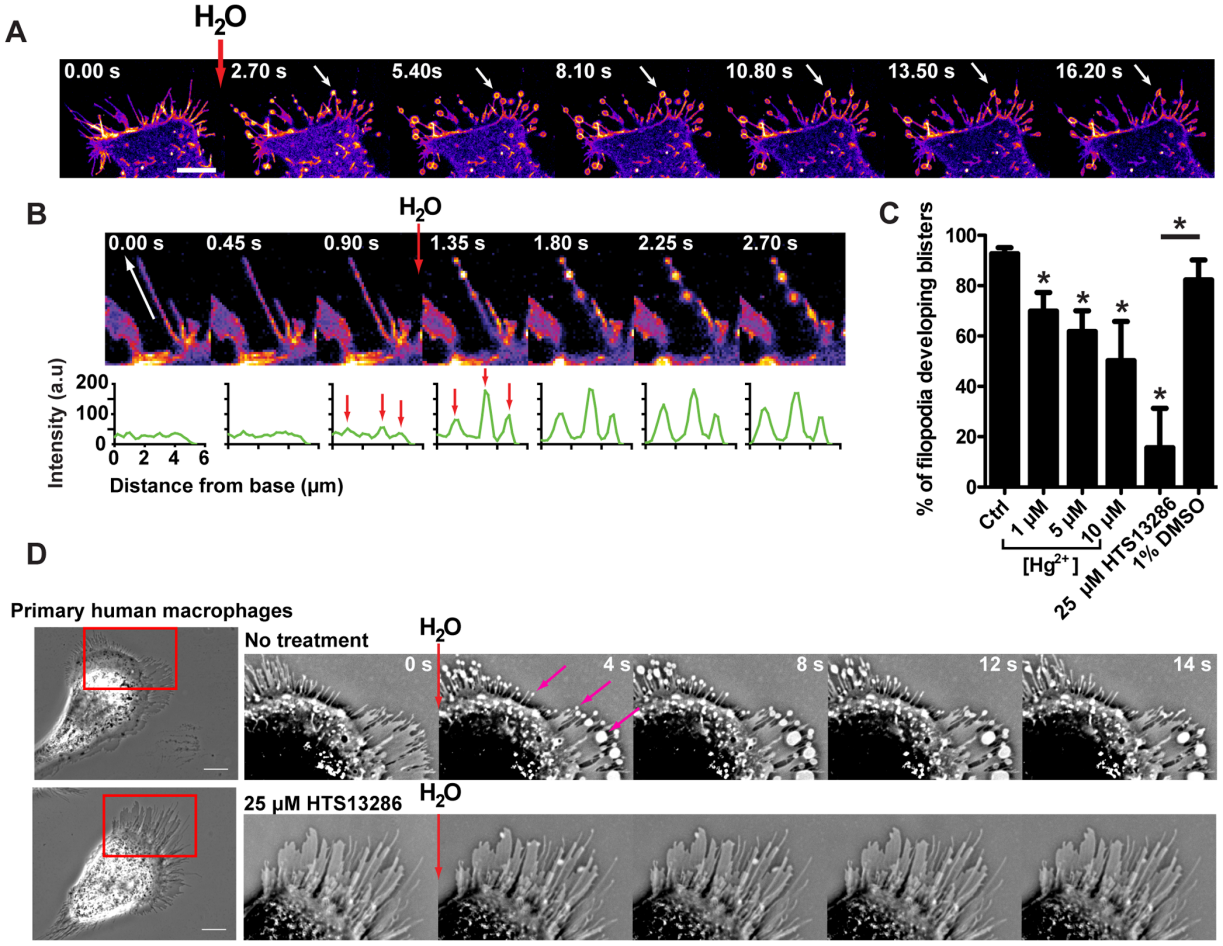
Addition of H_2_O to the medium causes filopodial bleb-like protrusions. (A) Confocal time-lapse montage of a HEK-293 cell stably overexpressing GFP-AQP9. During acquisition, 20 µl of H_2_O was added to the medium (2 ml) with a pipette directed towards the cell, yielding a rapid but transient reduction in local osmolarity. The images are pseudo-colored in fire scale to visualize variations in fluorescence intensity. White arrows are pointing towards a representative bleb-like protrusions formed during image acquisition. Scale bar 10 µm. (B, upper panel) An enlarged image of a single filopodium during acquisition, before and after the addition of H_2_O. The white arrow shows the direction and length of measurement presented in the lower panel. The images are linearly adjusted and pseudo-colored in fire scale to visualize variations in fluorescent intensity. (B, lower panel) Intensity profile plots measured along the filopodia as shown by the white arrow. The red arrows are pointing towards peaks in fluorescent intensity before and after the addition of H_2_O. (C) Quantification of the percentage of filopodia that developed filopodial bleb-like protrusions subsequent to the addition of 20 µl of H_2_O after pre-treatment with AQP9-inhibitors. HEK-293 cells overexpressing GFP-AQP9 were pretreated with 1, 5 and 10 µM Hg^2+^ or with 25 µM of HTS13286. Control cells represents untreated HEK-293 cell overexpressing GFP-AQP9. Data is presented as mean (±SEM, n = 4–7 experiements/group). (D, left panel) Phase contrast images of primary human macrophages. The cell in the lower panel is treated with 25 µM of the novel AQP9 inhibitor HTS13286. (D, right panel) Cropped and inverted time lapse montage of the cells displayed in the left panel. During image acquisition 20 µl of H_2_O was added to the medium (2 ml). Magenta arrows are pointing towards filopodial bleb-like protrusions. Scale bar 10 µm.

To seek evidence for the functionality of water channels in filopodia of AQP9-GFP expressing cells, we added a small volume of water viz. 20 µl H_2_O to 2 ml of medium directed towards the cells. This yielded a rapid but transient reduction in osmolarity and resulted in a rapid transformation of the architecture of the filopodia, and formation of GFP-AQP9-enriched bleb-like protrusions (arrows in Fig. 3A, Video S3).

The experiments were repeated with addition of buffer as a control and to exclude possible influences of external pressure from the pipette, which did not have any observable effects (Video S3). Moreover, the bleb-like protrusions were formed on the filopodia at sites with the highest initial GFP-AQP9 fluorescent intensity, indicating a water influx-driven mechanism. Fluorescence intensity continued to increase during bleb-like formation, indicating further AQP9 accumulation at these sites (Fig. 3B). In addition, the percentage of filopodia that formed bleb-like protrusions decreased from 92(±2.4)% to 70(±7.4), 62(±8.4) and 50 (±15.6;mean±SEM, p<0.05)% upon inhibition of AQP9 with 1, 5 or 10 µM Hg^2+^, respectively (Fig. 3C, n = 6–7 experiments/group). Moreover, addition of 25 µM of HTS13286, a novel specific AQP9 inhibitor [53], decreased the percentage of filopodial bleb-like protrusions from 82(±7.9)% (vehicle control) to 16(±15.6)% (Fig. 3C, p<0.05, n = 4–5 experiments). To further investigate whether this effect occurred in cells with endogenous AQP9 [54], [57], we imaged primary human macrophages during the addition of H_2_O. Indeed, these cells also formed filopodial bleb-like protrusions (Fig. 3D upper panel). Again, the effect was almost completely inhibited after inhibition of AQP9 with HTS13286 (Fig. 3D lower panel, representative data from 1 out of 4 experiments yielding essentially identical results).

### Filopodial Elongation Precedes Actin Polymerization in AQP9-induced Filopodia

**Figure 4:**
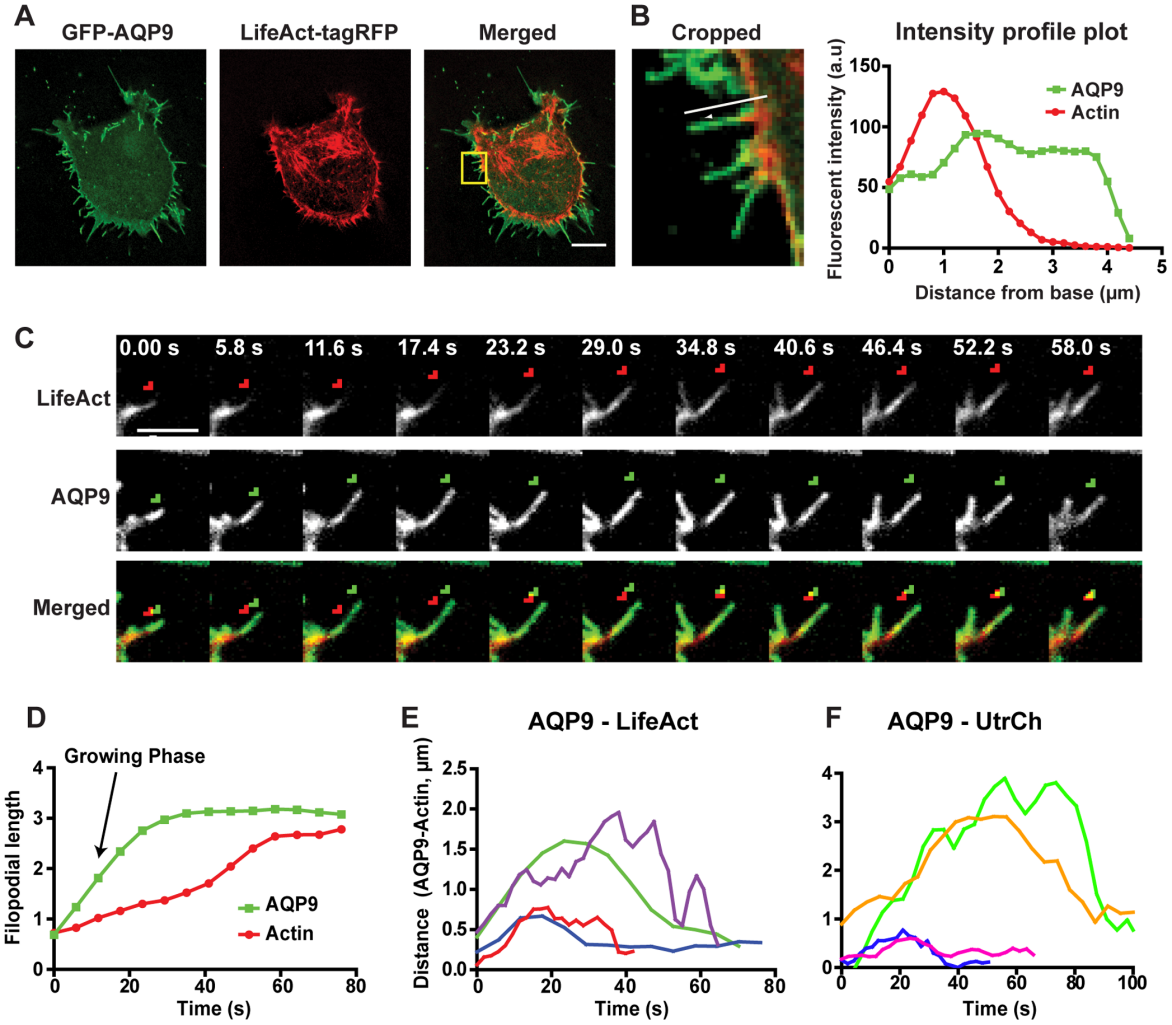
Interplay of filopodial actin and AQP9 reveals temporal changes in their relative distribution. (A) Representative confocal images of a HEK-293 cell stably overexpressing GFP-AQP9 and transfected with the actin-filament labeling probe tagRFP-LifeAct. Intensities have been adjusted linearly to visualize the relative distribution of both fluorophores. Scalebar 10 µm. (B, left panel) Enlarged area of the yellow box in A. Arrow shows the direction and filopodia subjected for measurement in the right panel. (B, right panel) Relative intensity profile plot of the filopodia presented in the left panel. The data is presented as a moving average of 3 adjacent values. (C) A cropped confocal time-lapse montage of tagRFP-LifeAct (upper panel, red in lower panel) and GFP-AQP9 (middle panel and green in lower panel) distribution during filopodial growth. To highlight differences in spatial distribution, the tip of the actin filaments were labeled with a red arrow and the tip of the AQP9 fluorescent intensity is labeled with a green arrow. Linear intensities have been adjusted to visualize the relative distribution of both fluorophores. (D) A representative image showing the analysis of the relative distribution of GFP-AQP9 and tagRFP-LifeAct along the length of a filopodium during growth. The data is presented as a moving average of 3 adjacent values. (E) Illustration of the space between the filopodial tip in the GFP channel versus the RFP channel in four filopodia imaged in HEK-293 cell overexpressing both GFP-AQP9 and tagRFP-LifeAct. The initial point represents the initiation of growth, and the terminal point represents the end of growth. Each colored line represents the distance between AQP9 and actin in individual filopodia. The data is presented as a moving average of 3 adjacent values.(F) Similar analysis as in E, using mRFP-UtrCH showing the gap between AQP9 fluorescence and actin filaments was not due to interference of polymerization or loss of binding by the actin labeling probe in E.

Since filopodia are assumed to contain long and tightly bundled actin filaments, we investigated the actin structure in AQP9-induced filopodia. Upon transient expression of tagRFP-LifeAct in HEK-293 cells with stable GFP-AQP9 overexpression, we did indeed observe actin in these structures (Fig. 4A–D) but it did not extend all the way to the filopodial tip during protrusion (Fig. 4B), Moreover, the space between the filopodial tip and actin appeared to vary during growth of the filopodia and the distance between tagRFP-LifeAct and GFP-AQP9 was largest during rapid growing phases (Fig. 4C and D). After the filopodia reached their apparently final length, the actin polymerized into the gap, thereby closing the space between actin and AQP9 (Fig. 4D). This effect was even more evident after analysis of the distance between actin and AQP9 in filopodia from the initiation of a rapid extension phase to the termination of growth (Fig. 4E). The formation of the filopodia differed in speed and length (data not shown), but the extension phase always started with a large distance between AQP9 and actin and subsequently decreased towards termination of growth (Fig. 4E). To corroborate these findings we repeated the experiments using the calponin homology domain of utrophin coupled to RFP (RFP-UtrCH) to visualize actin dynamics [55]. Indeed, essentially identical results were obtained for the space between GFP-AQP9 and RFP-UtrCH during filopodial growth (Fig. 4F).

### AQP9-induced Filopodial Dynamics and Rigidity Depends on Actin Remodeling

**Figure 5:**
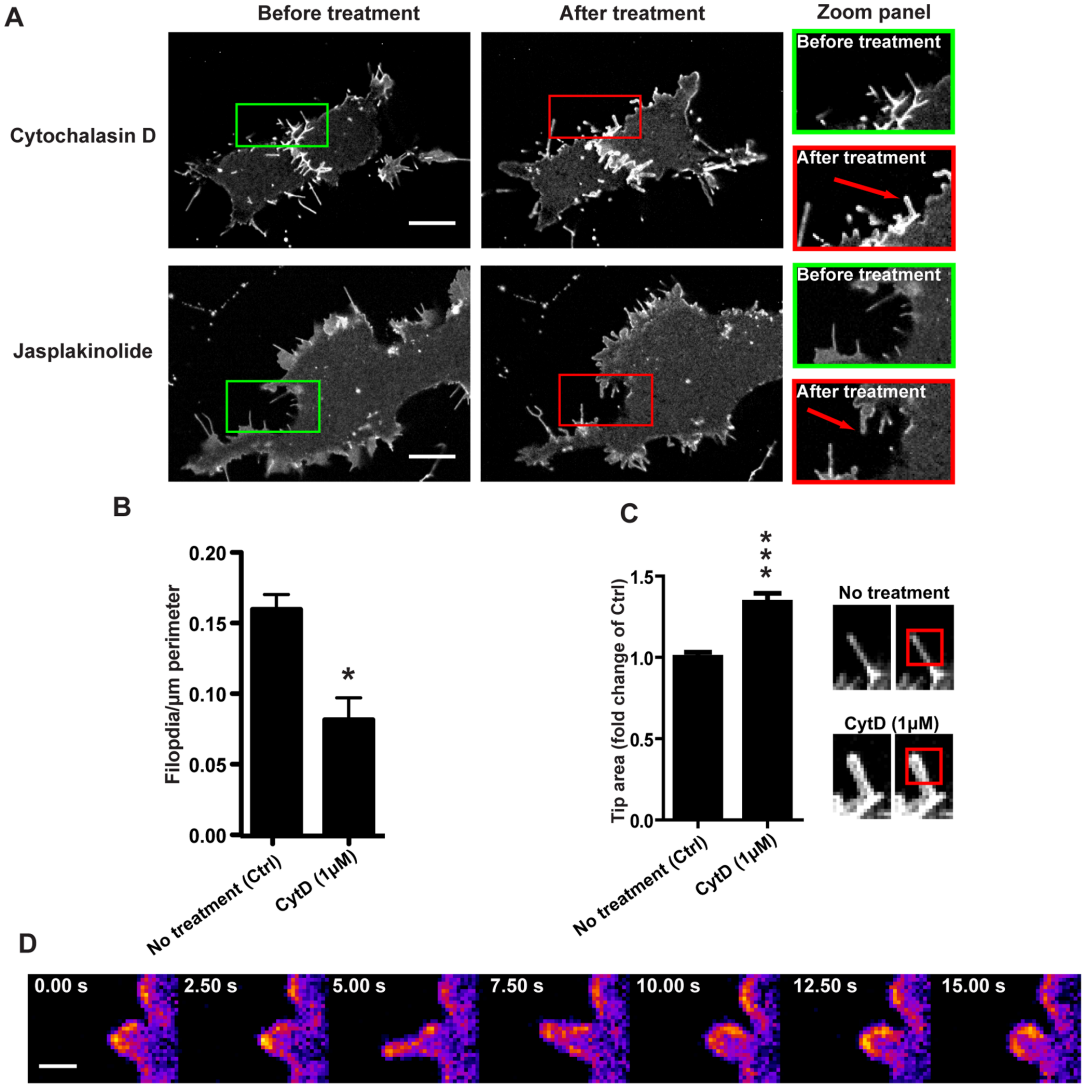
Disruption of actin dynamics inhibits the formation of new filopodia. (A) Representative confocal images of HEK-293 cells stably overexpressing GFP-AQP9 before and 15 min after treatment with 1 µM Cyt D or 500 nM Jasplakinolide. The red arrows point towards distended filopodia. Scalebar 10 µm. (B) Quantification of peripheral filopodia before, and 10–15 min after treatment with 1 µM Cyt D. The data is presented as mean (±SEM, n = 5–43 cells/group). (C, left panel) Quantification of the relative filopodial tip area of GFP-AQP9 expressing cells before (Ctrl), and 15 min after the addition of 1 µM Cytochalsin D. The filopodial tips are defined by the fluorescent area occupied in a 2×2 µm ROI of the filopodial tips. The data is presented as mean (±SEM) of fold change compared to untreated cells (Ctrl; n = 13–22 filopodia/group).(C, right panel) Representative examples of a filopodia before and after treatment with Cyt D. The red box illustrates the area of measurement for the data presented in the left panel. (D) A confocal time-lapse montage of GFP-AQP9 fluorescence, pseudo-colored in fire scale, in HEK-293 cells 10 min after treatment with 10 µM Cytochalsin D. The images illustrate a bleb-like protrusion that recoils back towards the cell body after treatment with actin dynamics inhibitors. The linear intensity is adjusted to visualize differences in fluorescence intensity. Scalebar 1 µm.

To assess the role of actin filament formation and stability of filopodia, we investigated the effect of inhibitors of actin dynamics. Upon treatment with 1 µM Cytochalasin D (Cyt D) or 500 nM Jasplakinolide (Jasp) several filopodia were lost (Fig. 5A and B, n = 5–43 cells/group, p<0.05) being 0.16 (±0.01) filopodia/µm perimeter in untreated cells and 0.08 (±0.02; mean±SEM) filopodia/µm perimeter treatment with 1 µM Cyt D. Although some filopodia were still present, the formation of new structures and the dynamics of protrusions were inhibited (Video S1). Furthermore, the remaining filopodia often lost their rigidity and typically pointed architecture (Video S1, red arrows, Fig. 5A red arrows and C, n = 13–22 filopodia/group, p<0.0001, fold increase 1.65±0.06; mean ± SEM). After addition of Cyt D some bleb-like protrusions were formed, which appeared to recoil back towards the cell body (Fig. 5D).

### A Sustained Osmotic Gradient Affects the Size, and Location of the Bleb-like Protrusions

**Figure 6:**
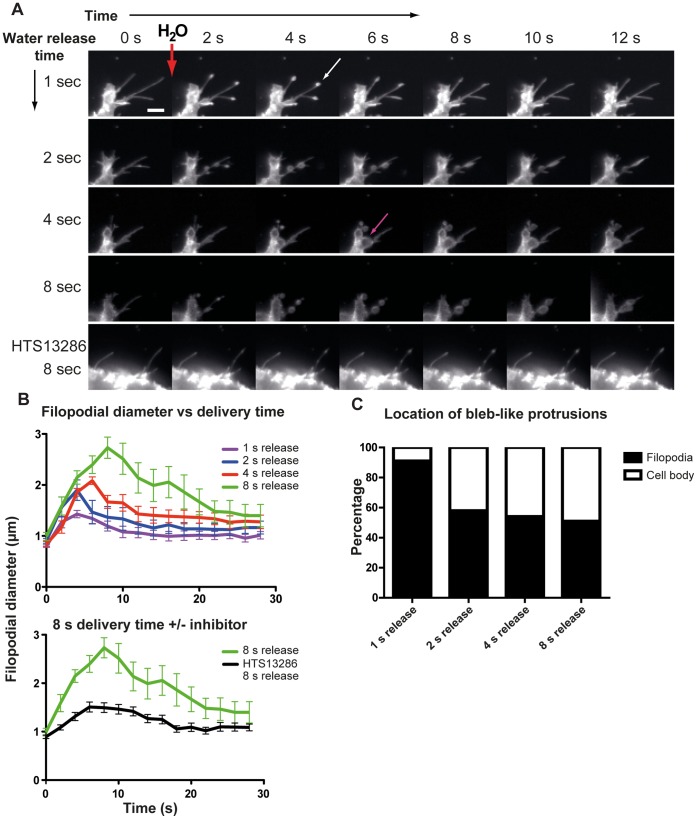
Size and location of H_2_O-induced bleb like protrusions. (A) Time lapse montage of the formation of filopodial bleb-like protrusions. H_2_O was delivered with a micropipette in close proximity to the cell. The pressure (4000hPa) was applied to the micropipette for 1, 2, 4 or 8 s to the same cell. The white arrow is pointing toward a filopodial bleb-like protrusion. Magenta arrow is pointing toward a bleb-like protrusion originating from the cell body. (A, lower panel) Following an 8 s localized water release in close vicinity of a cell that was pre-treated with 25 µM HTS13286 no bleb-like formations were observed. Scale bar 5 µm. (B) Quantification of the filopodial diameter at the site of the bleb-like protrusion. Time 0 equals the image before localized H_2_O release. The lower graph shows the mean diameter for 8 s water release to cells untreated (green line), or treated with 25 µM of HTS13286 (black line).The data is displayed as mean±SEM, n = 8–18 filopodia/water release period. (C) Quantification of mean percentage of bleb-like protrusions originating from filopodia or the cell body after 1, 2, 4 or 8 s injection of micropipette-delivered H_2_O (n = 3 experiments).

To investigate whether an osmotic gradient could affect the size of the bleb-like protrusion, we placed a micro-injector in close proximity of the cells in focus and looked for their morphology after such localized release of minute volumes of H_2_O for different periods of time. Indeed, the size increased with prolonged water release (Fig. 6A and B), with diameters of 1.43 (±0.07), 1.90 (±0.20), 2.09 (±0.07) and 2.73 (±0.21) µm (mean ± SEM; n = 8–18 filopodia/group) after 1-, 2-, 4- and 8-s releases, respectively (Fig. 6B, Video S4). These figures correspond to an average increase by 64, 118, 139 and 214% of the filopodial diameter. Furthermore, pre-treatment with the AQP9 inhibitor HTS13286 blocket this effect strongly, yielding a filopodial diameter of 1.51 (±0.1µm; mean ± SEM, n = 12 filopodia) and a relative increase of 73% after an 8-s treatment, i.e. comparable to 1-s release without the inhibitor (Fig. 6A and B lower panel). Moreover, the proportion of bleb-like protrusions expanding from the cell body (Fig. 6A magenta arrow) increased along with prolonged water release periods starting at 9% for 1-s and increasing to 42, 46 and 49% for 2-, 4- and 8-s pulses, respectively (Fig. 6C, Video S4, n = 3 experiments).

### AQP9 Concentration and Membrane Bleb Formation

**Figure 7:**
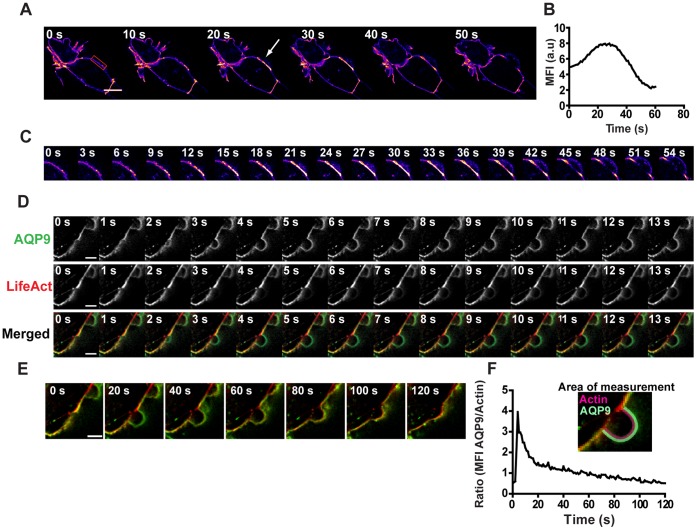
Water fluxes across AQP9 induce blebs. (A) A confocal time-lapse montage of HEK-293 cells stably overexpressing GFP-AQP9. The images are linearly adjusted and pseudo-colored in fire scale to visualize fluctuations in fluorescence intensity. The arrow is pointing towards GFP-AQP9 accumulation in the membrane Scalebar 10 µm. The red box represents the area of measurement for MFI in (B). A smoothing filter was applied to this image to reduce background. (B) MFI-measurement of the blebbing membrane throughout the time-lapse. The area of measurement is presented in (A). (C) Zoom in panel of the blebbing membrane presented in (A). (D) Representative confocal time-lapse montage of a blebbing HEK-293 cell expressing both GFP-AQP9 and tagRFP-LifeAct. Scalebar 5 µm. (E) The same montage as presented in (D) showing a longer acquisition time to illustrate the complete lifetime of the same bleb. (F) Ratio measurements obtained from the sequence presented in (E). The ratio is measured as AQP9 MFI in the bleb membrane divided by submembraneous actin MFI showing a rapid initial increase in ratio due to the absence of actin during bleb formation. The ratio subsequently decreases due to increasing actin fluorescence in the bleb. The insert is a representative image showing the area of measurement for AQP9 and actin.

Blebs are generally assumed to be induced by an acto-myosin-dependent contraction that increases the intracellular hydrostatic pressure tearing the membrane apart from the cortical actin [56]. We found that AQP9 accumulated at the membrane before unprovoked blebbing (Fig. 7A–C, Video S5). Incidentally, actin was initially not present in these blebs but appeared gradually during bleb stabilization and retraction (Fig. 7D–F). This was confirmed by the ratio between GFP-AQP9 and tagRFP-LifeAct intensity in the bleb over time (Fig. 7F).

## Discussion

The aim of this study was to investigate the localization and dynamics of the water channel AQP9 in relation to actin dynamics in the development of filopodia and blebs. We found that cells transfected with GFP-AQP9 developed numerous filopodial protrusions compared to the membrane-labeling control vector (Fig. 1A and B), and it furthermore localized and accumulated preferentially in these protrusions (Fig. 1 C–F). The water channels were indeed functional, since minute volumes of H_2_O added to GFP-AQP9 overexpressing cells induced bleb-like protrusions at sites of high channel concentration, viz. in the filopodia (Fig. 3A–C). This was also seen in primary human macrophages that endogenously express AQP9 [57], suggesting that localized addition of external water is sufficient to locally deform and expand the membrane and that this effect is enabled or facilitated by the presence of water channels (Fig. 3D). Physiologically, this assigns a new potential role to filopodia as sensors, rapidly responding to osmotic changes to relieve the cell body from osmotic stress. Previous studies have demonstrated that expression of AQPs facilitates the rate of cell migration and that there is an accumulation of AQPs in the leading edge of migrating cells [48]–[50]. An increased water flux at these sites could enable a pressure-driven forward push of the membrane and facilitate diffusion and polymerization of actin monomers [37], [39]. Indeed, we found that actin polymerization occurred subsequently to filopodial induction in these cells (Fig. 4A–F), and there was also a submembraneous space between AQP9 and actin during filopodial growth, which was gradually filled with actin after termination of elongation (Fig. 4C–F). We cannot completely rule out that the filopodial tip may contain some F-actin, but it is unlikely that it at such a low concentration, below the detection limit, could push the membrane to generate filopodia. It has previously been proposed that G-actin monomers needed at sites of rapid actin polymerization are provided from the cell body via a hydrodynamic flow [58], which could explain the lag in actin polymerization observed in Figs. 4C–F. Tentatively, the pressure generated beneath the membrane from water fluxes through the membrane, will gradually equilibrate upon filopodia formation and allow G-actin to reach the barbed ends of the filament. In addition, the water should dilute the gel-like cytoplasm and thereby facilitate G-actin diffusion at these sites.

Here we observed that inhibition of actin dynamics dramatically blocked the filopodial activity (Video S1). However, in these instances the cell still generated small bleb-like protrusions that recoiled back towards the cell body. We believe that these protrusions represent failed attempts to induce filopodia due to the absence of polymerizing actin. Svitkina and co-workers recently reported that expression of the I-BAR domain from IRSp53 can induce filopodia void of actin [36]. They proposed that actin filaments may not always drive the protrusions but rather provide mechanical support, which is consistent with our results showing that actin appeared crucial for the long term integrity and stability of these structures (Fig. 5A–D, Video S1).

Addition of minute volumes of H_2_O in close proximity of the cells elicited spherical filopodial membrane protrusions, with increasing diameter and correlating with the amount of water added, they also appeared on the cell body (Fig. 6). Thus, membrane blebs seem to reflect the magnitude and site of the osmolarity change. Indeed, a link between AQPs and blebs have previously been discussed [59]. Physiologically, such an effect could be potentiated by accumulation of AQPs. Incidentally, we found an increased concentration of AQP9 before bleb formation in HEK-293 cells overexpressing GFP-AQP9 and undergoing unprovoked blebbing (Fig. 7A–C). These structures were initially void of actin (Fig. 7D–F) [41], [60]. Furthermore, there is evidence that: (i) the fluid within the bleb is diluted in relation to the cytoplasm, indicating influx of water from the extracellular space [49], [61], and (ii) hyper- and hypo-osmolar solutions decrease and increase the bleb size, respectively [42]. Charras et al., (2005), observed that the total cell volume remained fairly constant during blebbing, and therefore suggested that the fluid in the bleb might primarily come from the inside of the cell [42]. However, a localized influx of water through concentrated AQP9s would provide a local pressure between the membrane and the cortical cytoskeleton at the site of bleb induction, without significantly affecting the total cell volume. Moreover, the gel-like cytoplasm might limit the diffusion of water and thereby act as a barrier, enabling the pressure to transiently separate the membrane and cytoskeleton from each other**.** An apparent AQP9 accumulation always correlated with subsequent bleb development, but the center of the bleb did not precisely coincide spatially with the highest concentration (Fig. 7D and E). Albeit it seems to depend critically on water influx, it is likely affected by for instance myosin II contractions and the cortical actin structure [42], [43].

**Figure 8:**
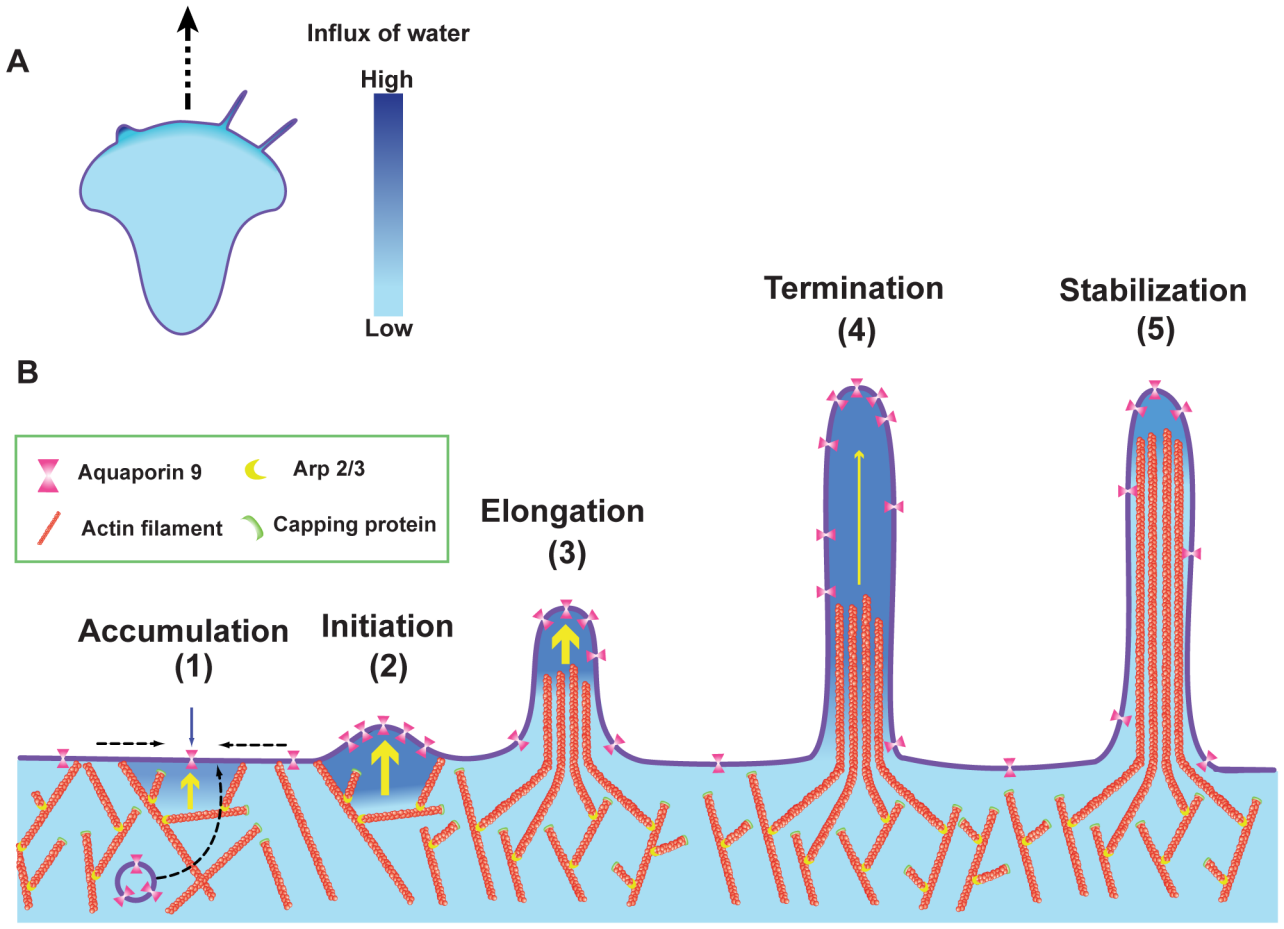
Model for AQP9-induced membrane protrusion. (A) A migrating cell with lamellipodia, filopodia, and blebs where an increased influx of water corresponds to a darker blue tone. (B1) Local accumulation of AQP9 by vesicle transport and/or lateral membrane diffusion enables a localized increased influx of water across the cell membrane. The influx is driven by an osmotic gradient, likely created by the transmembrane ion distribution (not shown). (B2) The rapid influx of water creates a localized hydrostatic pressure between the membrane and the cytoskeleton pushing the membrane outwards, thus initiating a membrane protrusion. (B3) The influx of water increases the hydrostatic pressure locally. In parallel, actin polymerization is promoted by the exposure of previously membrane-anchored barbed ends and the rapid diffusion of actin monomers in the now diluted, less viscous cytoplasm leading to an elongating filopodium. (B4) Then the rapid water-induced elongation reaches a critical distance from the actin, resulting in termination of the filopodial elongation likely due to equilibration of the water along the filopodium and loss of counter-pressure obtained from the actin cytoskeleton. (B5) The rate of the actin polymerization catches up with the water-induced protrusion and thereby stabilizes the structure. Based on the rate of water flux and equilibration, the filopodium can either protrude once more, or remain at its present length.

We believe that the driving force behind water-induced membrane protrusions relies on micro-osmotic gradients that enable water to rapidly flow across the membrane. As reviewed by Schwab and co-workers [59], such a gradient is probably not attributed to a single ion channel but rather a network of several ion transporters acting together as a migration-associated “transportome”, that localizes in close vicinity of water channels [59]. Thus, it is tempting to speculate that an interplay of these transporters and AQPs facilitate protrusion formation. Our findings can be summarized in a hypothetical model for how AQP9-induced water fluxes promote the formation of membrane protrusion (Fig. 8A). This is facilitated by accumulation of water channels by vesicle transport and/or lateral membrane diffusion (Fig. 8B). The influx of water builds up a pressure between the membrane and the cytoskeleton and forces the membrane to protrude outward and dilutes the submembraneous gel-like cytoplasm, which allows for rapid actin polymerization into the newly created gap. With a rapid influx of water, the rate of the filopodial protrusion first outpaces the actin polymerization creating a space between the membrane and the actin cytoskeleton. This will however soon equilibrate but temporarily helps actin filaments to polymerize into the protruding structure, while the water simultaneously equilibrates along the length of the filopodia (Fig. 8B). The protrusive force of AQP9-induced filopodia is limited by the size of the water flux and thus the osmotic gradient, but also by the cytoskeletal and membrane rigidity and the cellular contractile forces.

Furthermore, the formation of bleb-like protrusions in AQP9-enriched filopodia upon water addition indicate that they may serve as osmo-sensors, protecting the cells from osmotic stress. In conclusion, we show that water fluxes through AQP9 channels initiate membrane protrusions thereby directing actin polymerization and allowing the cells to change morphology and motility.

## Materials and Methods

### Ethics Statement

This study was conducted in accordance with the Declaration of Helsinki. Human blood was collected at the blood bank at Linköping University Hospital by employees at the blood bank division. A written consent for research use of donated blood was obtained from all donors. Since blood donation is classified as negligible risk to the donors and since only anonymized samples were delivered to the researchers, the research did not require ethical approval according to paragraph 4 of the Swedish law (2003∶460; http://www.lagboken.se/dokument/Lagar-och-forordningar/4060/Lag-2003_460-om-etikprovning-av-forskning-som-avser-manniskor?id=64991 ) on Ethical Conduct in Human Research.

### Vectors

For the generation of a stably GFP-AQP9 overexpressing cell line, the lentiviral expression system Lenti-X™ (Clontech laboratories inc, Mountain View, CA) was used. For transient transfections, the vector backbone from pEGFP-C1 (Clontech) was used. To label the membrane pAcGFP1-Mem was transfected into cells. We acknowledge the following kind gifts: the tagRFPt-LifeAct vector to visualize actin in living cells was a kind gift from Professor Theodorus Gadella (University of Amsterdam, Amsterdam, The Netherlands; [62], [63], the GFP-MyosinX construct from Professor Staffan Strömblad (Karolinska Institutet, Stockholm, Sweden; [64], the pGex-BAIAP2 vector from Professor Anne Brunet, (Stanford University, Stanford, CA, Addgene plasmid #31675; [65] and mRFP-UtrCH vector from Professor William M. Bement (University of Wisconsin-Madison, Madison, WI; Addgene plasmid #26739; [55].

### Cell Culture

HEK-293 cells (ATCC, Teddington, UK) were grown in Dulbeccos Modified Eagles Medium (DMEM) supplemented with 10% fetal bovine serum (FBS), 100 µg/ml streptomycin, 100 U/ml penicillin, 1 mM Sodium Pyruvate and 2 mmol L-glutamine (all obtained from GIBCO BRL/Invitrogen Carlsbad, CA, USA). The medium was changed every 2–3 days, and the cells were kept at sub-confluency (below 80%). Cells infected with lentivirus were cultured in the presence 1 µg/ml Puromycin (Sigma-Aldrich, St Louis, MO) until a 100% stable transfection was reached.

### Plasmid Construction and Viral Production

cDNA encoding human AQP9 was prepared as described previously in [38] and subsequently cloned into the lentiviral vector pLvx-AcGFP-C1 (Clontech) with the use of Bsp119I and BamHI (Fermentas, Thermo Scientific, Sunnyvale, CA) restriction sites. Retroviral particles were produced in GP-2 293 packaging cells (Clontech) for 48 h after transfection with the lentiviral vector together with Lenti-X HT packaging mix according to the manufactureŕs protocol. The virus-containing supernatant was centrifuged for 10 min at 500 g to spin down detached cells and the supernatant was subsequently aliquoted and stored at –70°C.

For transient transfections of GFP-AQP9 the plasmid pEGFP-C1-AQP9, previously described in [38] was used. To visualize actin in living cells, the plasmid ptagRFPt-LifeAct was transfected into cells. The plasmid ptagRFP-AQP9 was constructed by PCR amplification of tagRFP from ptagRFP-LifeAct with forward primer 5′-GCT AGC ACC GGT CGC CAC CAT-3′ and reverse primer 5′-AGA TCT CTT GTA CAG CAG TTT GCT-3′. The generated sequence was then ligated into pEGFP-C1-AQP9 after treatment of both the plasmid and insert with the restriction enzymes Nhe1 and BglII (Fermentas). The insert was fully sequenced to check for possible errors (Macrogen, Amsterdam, The Netherlands). For construction of the ptagRFP-BAIAP2 vector, pGex-BAIAP2 and ptagRFP-AQP9 were treated with the restriction enzymes EcoRI and XbaI (Fermentas). Then the cDNA encoding BAIAP2 was ligated into the linearized ptagRFP vector to generate tagRFP-BAIAP2.

### Transfection and Infection

For transient transfections, vector DNA was transfected into cells with the aid of Turbofect transfection reagent (Fermentas) according to manufacturers protocol. In brief, a mixture of 1 µg of vector DNA, 2 µl of Turbofect and 100 µl of serum-free medium was allowed to equilibrate for 20 min and subsequently added to individual wells in a 6-well plate containing cells at 50–70% confluency. The cells were incubated for around 15 h, trypsinized and seeded into 35 mm glass-bottom culture dishes (MatTek corporation, Ashland, MA) and allowed to adhere for 24 h prior to the experiment. For viral infections, sub-confluent HEK-293 cells cultured in 10-cm dishes were infected with 200 µl of the lentiviral aliquots for 24 h. The cells were then washed in PBS and fresh media was added to the cells. After 24 hours, puromycin (Sigma) was added to the dish (1 µg/ml) for selection of infected cells.

### Isolation and Differentiation of Primary Human Macrophages

Primary human monocytes were isolated from whole blood obtained from healthy blood donors and differentiated to become macrophages essentially as described in [66]. In brief, whole blood was layered onto Lymphoprep (Axis Shield, Dundee, Scotland) and subsequently centrifuged for 40 min at 480×g, RT. The mononuclear cells were collected and washed three times in PBS, then cultured in DMEM supplemented with 1 U/ml penicillin, 10 µg/ml streptomycin (all obtained from GIBCO BRL/Invitrogen) 80 µM L-Glutamine and 10% normal human serum pooled from five donors (Blood Bank at Linköping University Hospital) and allowed to differentiate to become macrophages for 8 days.

### Imaging

Live cell imaging was carried out on an Axiovert 200 M (Zeiss, Jena, Germany) stage equipped with a mercury short-arc lamp (HXP120c; Zeiss) and structured illumination-aperture correlation unit (VivaTome, Zeiss) and a filter set optimized for DAPI (not used), FITC (ex; 494/20–25, em; 536/40–25), and TexasRed (ex; 575/25–25, em; 628/40–25) in combination with a triple band dichroic mirror (436/514/604) or a filter set optimized for GFP (ex; 457/50–25, em; 525/50–25) and DsRed (ex; 556/20–25, em; 617/73–25) combined with a dual band dichroic mirror (493/574; all purchased from Zeiss). Both sets were suitable for visualization of GFP and RFP. For multichannel imaging, the images were acquired sequentially. The microscope was also equipped with a 100x (NA 1.4; Zeiss) 63x (NA 1.25; Zeiss) and 40x (NA 1.3;Zeiss) objectives. Phase contrast imaging was carried out with a 100x (NA 1,3, Ph; Zeiss) objective. The detector for this system was an Axiocam MRm CCD camera with a pixel size of 6.45×6.45 µm. All objectives were preheated to 37°C with an objective heater (Peacon, Erbach-Bach, Germany). Live cell imaging was carried out in Krebs-Ringer Glucose buffer supplemented with 1 mM Ca^2+^ (pH 7.3; Calcium-containing media; CCM) and the cells were kept on the microscope for <30 mins.

### Treatment with Disruptors of Actin Dynamics

HEK-293 cells stably overexpressing GFP-AQP9 were visualized before- and 10–15 min after the addition of 1 µM Cytochalasin D (Sigma) or 500 nM Jasplakinolide (Calbiochem, MERCK, Darmstadt, Germany) respectively. For time-lapse imaging, the cells were further subjected to a transient transfection with tagRFPt-LifeAct as previously described and a short confocal sequence was captured with the VivaTome before and 10 min after treatment with Cyt D or Jasplakinolide. The time interval between frames was set to 5 s and the integration time was individually set for each sample but was generally kept below 1 s.

### H_2_O Delivery and Bleb-like Protrusion Quantification

Addition of 20 µl of H_2_O during image acquisition was performed manually with a pipette directed towards the cells that were seeded in 2 ml CCM; as a control, 20 µl of CCM was added instead of water. During the experiment, GFP-AQP9 was visualized with the VivaTome. For H_2_O delivery to primary macrophages the same procedure was used but the image sequences were captured using phase contrast microscopy. To investigate the effects of AQP9 inhibitors on the development of bleb-like protrusions the cells were pre-treated for 15 min with 1, 5 or 10 µM Hg^2+^ or 25 µM HTS13286 (Maybridge, Cornwall, UK). As vehicle for HTS13286, 1% DMSO was used. For quantification, all filopodia that were visible before and after the addition of H_2_O were counted manually in ImageJ. Localized addition of water was done with a microinjection system consisting of a Femtojet and an InjectMan NI2 equipped with Eppendorf femtotips (Eppendorf, Hamburg, Germany). The release of water was applied for 1, 2, 4 or 8 s in close proximity of the cells. The pressure on the micropipette during water release was kept at 4000 hPa. Only filopodia located within a diameter of 75 µm from the micropipette were measured. The pixel size during measurements was 0.3 µm/pixel. The filopodial diameter was measured manually on images before and during the localized release of H_2_O. Quantification of the number of bleb-like protrusions located on filopodia versus the cell body was done manually after the release of H_2_O. The data are presented as the proportion (%) of bleb-like protrusions originating from the cell body or filopodia compared to different time intervals of localized release of H_2_O.

### Quantification of Filopodia

Cells were transfected with tagRFP-AQP9 or empty vector, together with GFP-Mem and seeded into glass bottom culture dishes (MatTek). Live cell images from an optical section located at the basal part of single cells were captured sequentially in both the GFP and RFP channels with the VivaTome. The camera integration time varied slightly between sequences but was generally kept shorter than 1 s. After image acquisition, the number of lateral filopodia/cell as well as the cell perimeter was quantified manually with ImageJ. To assess the effect of HgCl2, the images were acquired 30 min after pre-treatment with 1, 10 or 100 µM HgCl2. To quantify the amount of filopodia before and after fixation, 4% of paraformaldehyde in CCM was preheated to 37°C and added to the cells that were subsequently incubated for 20 min. The cells were then carefully washed twice in CCM before image acquisition.

### Quantification of Filopodial Tip Area in Cyt D-treated Cells

Images representing an optical section at the basal part of HEK-293 cells stably overexpressing GFP-AQP9 were captured with the VivaTome. The filopodial tip-area was quantified as the fluorescent area within a 2×2 µm region of interest (ROI) located at the filopodial tip. To exclude background fluorescence from the cell body or adjacent filopodia, only filopodia longer than 2 µm with a distance of more than 2 µm from adjacent filopodia were subjected to analysis. The data are presented as the tip-area for GFP-AQP9 expressing cells treated with Cyt D (1 µM) compared to untreated GFP-AQP9 transfected cells (Ctrl). The pixel size in the images was 0.2 µm/pixel.

### Quantification of AQP9 Localization in the Filopodial Membrane

Images of living cells transiently transfected with both tagRFP-AQP9 and GFP-Mem were captured with the VivaTome and analyzed with ImageJ. The mean fluorescent intensities in regions of interests in filopodial and sub-filopodial membrane were measured at the same location in both channels as shown in Fig. 1D.

### Quantification of Filopodial Growth

Cells stably overexpressing GFP-AQP9 were transfected with tagRFP-LifeAct or mRFP-UtrCH and allowed to adhere for 24 h in glass bottom culture dishes (MatTek). After adhesion, image sequences were acquired sequentially, but immediately after one another in the red and green channel with the VivaTome. The camera integration time was in general kept below 1.5 s/image which is well below the dynamics for filopodia formation. The time interval for image acquisition differed among the sequences ranging from 1.9 s to 5.8 s between frames (mean 3.5 s between frames). The total measurment varied from 2 to 7 min (mean 4 min) and total amount of images/sequence ranged from 37 to 143 (mean 85 images/sequence). Image analysis was carried out in ImageJ, where peripheral growth of filopodia was tracked by manually tracking the filopodial length over time. In the analyses, pixels within the cell body were allowed to saturate when assessing the lower concentration in the filopodia. Focus for the initial image was alternated between the red and green channel to account for potential differences in focal planes. Similarly, the initial image was alternated between the red and green channel. To adjust for small differences between images within a sequence the data is presented as a moving average of three time-points. To analyze the space between AQP9 and actin the same procedure was used and the length of actin fluorescence was subtracted from GFP-AQP9 fluorescence. Due to varying levels of GFP-AQP9 and tagRFP-LifeAct expression the camera pixels were occasionally binned 2×2. Thus, the pixel size was 0.1 or 0.2 µm.

### Analyses of AQP9 Intensity and the Relative Actin Content in Blebs

During the experiments, all cells were blebbing spontaneously. Optical sections of cells stably overexpressing GFP-AQP9 were captured with the VivaTome at 1 image/sec for a total period of less than 4 min. For imaging of GFP-AQP9 together with actin, the same procedure was used with cells that were transiently transfected with tagRFP-LifeAct. The camera integration time varied between samples and channels but was constantly kept below 200 ms. The pixel size in images of cell expressing only GFP-AQP9 was 0.1 µm whereas images of co-transfected cells had a pixel size of 0.3 µm. To measure GFP-AQP9 intensity fluctuations in the membrane during bleb development, a square ROI was placed at the blebbing site and the MFI within the box was measured throughout the sequence. For measurement of the ratio between AQP9 and actin, the MFI for AQP9 was measured as a line along the bleb membrane throughout the bleb whereas the location of measurement for actin MFI was as a similar line adjacent to the latter. A new line for intensity measurements was drawn in each image throughout the sequence because the shape and size of the bleb was constantly changing.

### Statistical Analyses

Generally, the data are presented as mean±SEM. For statistical analyses Students T-test was used for symmetrically distributed data where n>15 and non-parametrical Mann-Whitney for non-symmetrically distributed data or when n<15. The significance was rated * when p<0.05, ** when p<0.01 and *** when p<0.001.

## Supporting Information

Video S1 **Video of three HEK-293 cells stably overexpressing GFP-AQP9 (green) and transfected with tagRFP-LifeAct (red).** Images were acquired before- and 10 min after addition of either PBS (Ctrl), Cytochalasin D (1 µM) or Jasplakinolide (150 nM). By interfering with actin dynamics, the filopodial dynamics diminish completely. The linear intensity is adjusted to reveal the relative distribution of both fluorophores. The red arrows point toward typically bulky filopodia that fails to elongate after Cytochalasin D treatment. Scalebar 10 µm.
(AVI)

Video S2 **Confocal video of two stably GFP-AQP9 overexpressing HEK-293 cells subjected to addition of 1 or 10 µM Hg^2+^ during image acquisition.** The addition of Hg^2+^ is indicated with white arrows. The time between frames is 5 s. Scalebar 10 µm.
(AVI)

Video S3 **Confocal video of two stably GFP-AQP9 overexpressing HEK-293 cells subjected to a 20 µl addition of distilled H_2_O or CCM (Ctrl) during image acquisition.** The addition of H_2_O is indicated with white arrows. The time between frames is 0.4 s. Scalebar 10 µm.
(AVI)

Video S4 **Confocal video of a HEK-293 cell, stably overexpressing GFP-AQP9.** During acquisition the cell was subjected to localized release of H_2_O delivered with a micropipette. The white arrow illustrates the duration of H_2_O release. Time between frames is 2 s. Scalebar 10 µm.
(AVI)

Video S5 **Confocal video of a blebbing HEK-293 cells, stably overexpressing GFP-AQP9.** The video is pseudo-colored in fire scale and intensity adjusted linearly to clearly visualize AQP9 accumulation before bleb induction. The white arrows indicate examples of AQP9 accumulation followed by bleb formation. Time between frames is 10 s.
(AVI)
